# Residues of tetracyclines and β-lactams antibiotics induce carbonylation of chicken breast

**DOI:** 10.12688/f1000research.53863.1

**Published:** 2021-07-15

**Authors:** Johana Marquez, Albeiro Marrugo Padilla, Darío Méndez Cuadro, Erika Rodríguez Cavallo

**Affiliations:** 1 Analytical Chemistry and Biomedicine Group, University of Cartagena, Cartagena, Bolívar, 130001, Colombia; 2 Medical research group (GINUMED), Corporación Universitaria Rafael Núñez, Cartagena, Bolívar, 130001, Colombia

**Keywords:** Tetracyclines, β-lactams, carbonylation, proteins, meat

## Abstract

**Background:** Worldwide, chicken meat is widely consumed due to its low cost, high nutritional value and non-interference with religious or cultural beliefs. However, during animal husbandry chickens are exposed to many chemical substances, including tetracyclines and β-lactams, which are used to prevent and cure several infections. Some residues of these compounds may bioaccumulate and be present in chicken meat after slaughtering, promoting oxidative reactions.

**Methods:** In order to evaluate *in vitro* carbonylation induced by tetracyclines and β-lactams residues, a proteomic approach was used. For this, chicken muscle was individually contaminated with tetracyclines (tetracycline, chlortetracycline, oxytetracycline, and doxycycline) and β-lactams (ampicillin, benzathine penicillin, dicloxacillin and oxacillin) at 0.5, 1.0 and 1.5 times their maximum residue level (MRL). Then, sarcoplasmic, myofibrillar and insoluble proteins were extracted and their content were measured using the Bradford method. Protein carbonylation was measured using the 2,4-Dinitrophenylhydrazine alkaline method.

**Results:** Residues of tetracyclines and β-lactams induced *in vitro* carbonylation on sarcoplasmic, myofibrillar and insoluble proteins even at 0.5MRL concentrations ( *p<0.05*). When comparing the carbonylation induced by both antibiotics no differences were found ( *p>0.05*). Variables such as the partition coefficient (log P) and the concentration of these antibiotics showed a high correlation with the oxidative capacity of tetracyclines and β-lactams on chicken breast proteins.

**Conclusions**: This study shows that the presence of tetracyclines and β-lactams residues at MRLs concentrations promotes *in vitro *carbonylation on chicken breast proteins. Our results provide important insights about the impact of antibiotics on the integrity of meat proteins intended for human consumption.

## Introduction

Tetracyclines (TCs) and β-lactams (β-Lts) are widely used antibiotics in veterinary medicine for the treatment and prophylaxis of bacterial diseases affecting animals. ^1^ In poultry, these antibiotics are employed to treat diseases caused by gram-positive and gram-negative bacteria, and in some cases are also administered at sub-therapeutic concentrations in the feed to promote the animal’s growth and ensure productive performance. ^2, 3^ In this context, this extensive and often indiscriminate use of TCs and β-LTs in poultry can induce the presence of their residues in poultry products such as chicken breast and eggs; these residues can reach and be ingested by consumers, and promote negative effects on their health, mainly through antimicrobial resistance. ^1^

In order to protect to consumer, the European Commission and the Codex Alimentarius have stablished the maximum residue level (MRL) for antibiotics in products of animal origin. The MRL is defined as the highest level of residue of a pesticide or antibiotic that is legally tolerated in food and is not expected to cause harm to humans. Despite the regulation, several investigations have reported levels of TCs and β-LTs greater than their MRLs in chicken meat and other products derived from poultry farms, such as eggs; this could promote negative effects on consumers stated above. ^4, 5^

Despite the recurring presence of antibiotic residues in chicken meat and its derivatives, no investigations evaluating the effects of TCs and β-LTs at their MRLs on chicken meat components, including proteins, are being carried out, even though proteins are one of its most abundant constituents. In previous studies we have demonstrated that residues of these antibiotics at their MRLs are capable of inducing oxidative stress on biological models such as beef ^6^ and milk, ^7^ which could have a negative impact on food safety and quality. Protein carbonylation is currently recognized as one of the main biomarkers for oxidative stress in food, ^8^ and several publications have demonstrated its impact in chicken meat and the alteration of its functional properties such as solubility and digestibility. ^9, 10^ In addition, the effects promoted by the consumption of foods with carbonylated proteins, such as metabolic disorders and inflammatory processes at the intestinal level and even the promotion of carcinogenic processes, have also been demonstrated. ^11, 12^

Chicken meat is the most common product consumed in the word due its affordability, although chicken meat proteins are susceptible to suffer oxidative reactions which can be promoted by external factors as animal feed, slaughter, processing and storage. Thus, the objective of this study is to evaluate the *in vitro* carbonylation induced by tetracyclines and β-lactams residues on chicken muscle proteins.

## Methods

### Chemicals and reagents

Primary standards of Ampicillin trihydrate (99.0%, AMP), Benzathine penicillin G tretrahydrate (97.2%, PNG), Dicloxacillin sodium hydrate (99.0%, DCL), Oxacillin salt hydrate (99.0%, OXA), Tetracycline hydrochloride (97.7%, TC), Doxycycline hyclate (98.7%, DXC), Chlortetracycline hydrochloride (94.6%, CTC) and Oxytetracycline hydrochloride (95.0%, OTC) were supplied by Dr. Ehrenstofer GmbH (Germany). The 2,4-Dinitrophenylhydrazine, potassium phosphate, sodium azide, hydrochloric acid, sodium bicarbonate and calcium chloride were purchased from PanReac (Barcelona, Spain). Analytical grade methanol, sodium hydroxide EMSURE (99%), biotechnological grade sodium chloride, 2-mercaptoethanol, and coomassie blue brilliant G250 (CBB) were purchased from Merck (Darmstadt, Germany). Bovine serum albumin (BSA) free of fatty acids (98%) was supplied from Sigma Aldrich (San Luis, USA). Water was purified with a Milli-Q system (Millipore, Bedford, MA, USA).

### Preparation of antibiotics solutions

Individual stock solutions of tetracyclines and β-lactams (100 μg·mL ^-1^) were prepared with milli-Q water and methanol, respectively. For the sample’s contamination with the antibiotics of interest, serial dilutions of the stocks were made until obtaining an antibiotic concentration of 10 μg·mL ^−1^ (working solution), in order to avoid sample protein precipitation during the sample preparation process. ^13^

### Sample preparation and contamination

Chicken breast samples were purchased from a local market in Cartagena, Colombia. They were stored in plastic polyethylene bags, transported to the laboratory maintaining cold chain (4°C), cut and cleaned to remove visible connective tissue and then, mechanically homogenized using a blender (Powergen by Fisher). Then, one-gram replicates of homogenized samples were collected in falcon tubes and stored at -20°C until further assays. This procedure was described by Marquez *et al*. (2020). ^13^

Chicken breast aliquots were randomly divided in control samples and samples to contaminate with tetracyclines and β-lactams. Then, samples were contaminated individually with 200 μL of each antibiotic working solutions until reaching final concentrations of 0.5, 1.0 and 1.5 MRL ( Table 1), followed by vortexing for 30s and then incubation for 1 h at room temperature in the dark. ^14^ All assays were carried out with three replicates.

### Extraction of chicken breast proteins

To obtain the sarcoplasmic, myofibrillar and insoluble proteins, the method developed by Marquez *et al.* (2020) was employed. Briefly, homogenized samples were mixed with 10 mL of low ionic strength buffer (0.05 M K _3_PO _4_, 1 mM NaN _3_, 2 mM EDTA, pH 7.3), then, vortexing at 3,000 rpm for 3.5 minutes and centrifugation at *11,150 xg 10 minutes* and 1 °C were carried out. The supernatant (sarcoplasmic proteins) was collected and refrigerated, and the pellet was resuspended with 5 mL of low ionic strength buffer, centrifuged and the supernatant was unified with the sarcoplasmic proteins fraction initially obtained. The remaining pellet was resuspended with 5 mL of high ionic strength buffer (0.55 M KCl, 0.05 M K _3_PO _4_, 1mM NaN _3_, 2 mM EDTA, pH 7.3) and the same vortexing and centrifugation steps were realized.). The new supernatant (myofibrillar proteins) was collected and also kept refrigerated at 4°C. The final pellet was resuspended in 0.15 M KCl to obtain the insoluble proteins. ^13^ Protein concentration was determined by the Bradford method (Coomassie blue brilliant/ethanol/phosphoric acid). For this, a calibration curve was realized using standard solutions of bovine serum albumin at concentrations between 0.0625 and 1.0 mg·L ^−1^, which were put in microplates with the samples and the Braford reactive. Then, the absorbance was measured at 595 nm. ^15^

**Table 1.** Tetracyclines and β-Lactams concentrations at 0.5, 1.0 and 1.5 MRL.

Tetracyclines	0.5 MRL ^*^	1.0 MRL ^*^	1.5 MRL ^*^
Tetracycline	50	100	150
Chlortetracycline	50	100	150
Oxytetracycline	50	100	150
Doxycycline	50	100	150
**β-Lactams**			
Ampicillin	25	50	75
Benzathine penicillin	25	50	75
Dicloxacillin	150	300	450
Oxacillin	150	300	450

* The concentration are expressed as μg. Kg ^-1^ chicken breast.

### Determination of carbonyl content in proteins samples

Protein carbonyl content was measured according to Mesquita *et al.* (2014) with some modifications described by Marquez *et al*. (2020). ^13, 16^ For this, 300 μL of 2,4-dinitrophenylhydrazine (DNPH, 10 mM in 0.5M H _3_PO _4_) was added to the same volume of protein solution (150 μg) and incubated in the dark for 10 minutes. Next, 160 μL of this solution was placed in a 96-well plate and 40 μL of 6M NaOH were added and incubation for 10 minutes was carried out. The change of colour was measured at 450 nm (FLUOstar Omega spectrophotometer, BMG-Lab Tech). The protein carbonyl content was calculated using the DNPH molar extinction coefficient corrected for microplates (ε = 11154 μM ^-1^.cm ^-1^). Protein carbonylation was expressed as nmol of carbonyls·mg ^-1^ of protein.

### Statistical analysis

Values are reported as mean ± SEM of three independent determinations. To assess the effect of antibiotics concentration on protein carbonylation, a unifactorial ANOVA with three levels and blank at 95% confidence was performed ( GraphPad Prism V5.01). Multiple comparisons of the means were made using the Tukey adjustment when the ANOVA was significant ( *p* < 0.05) for control and contaminated samples.

## Results

### Carbonylation induced by antibiotics on chicken breast proteins

**Figure 1.**
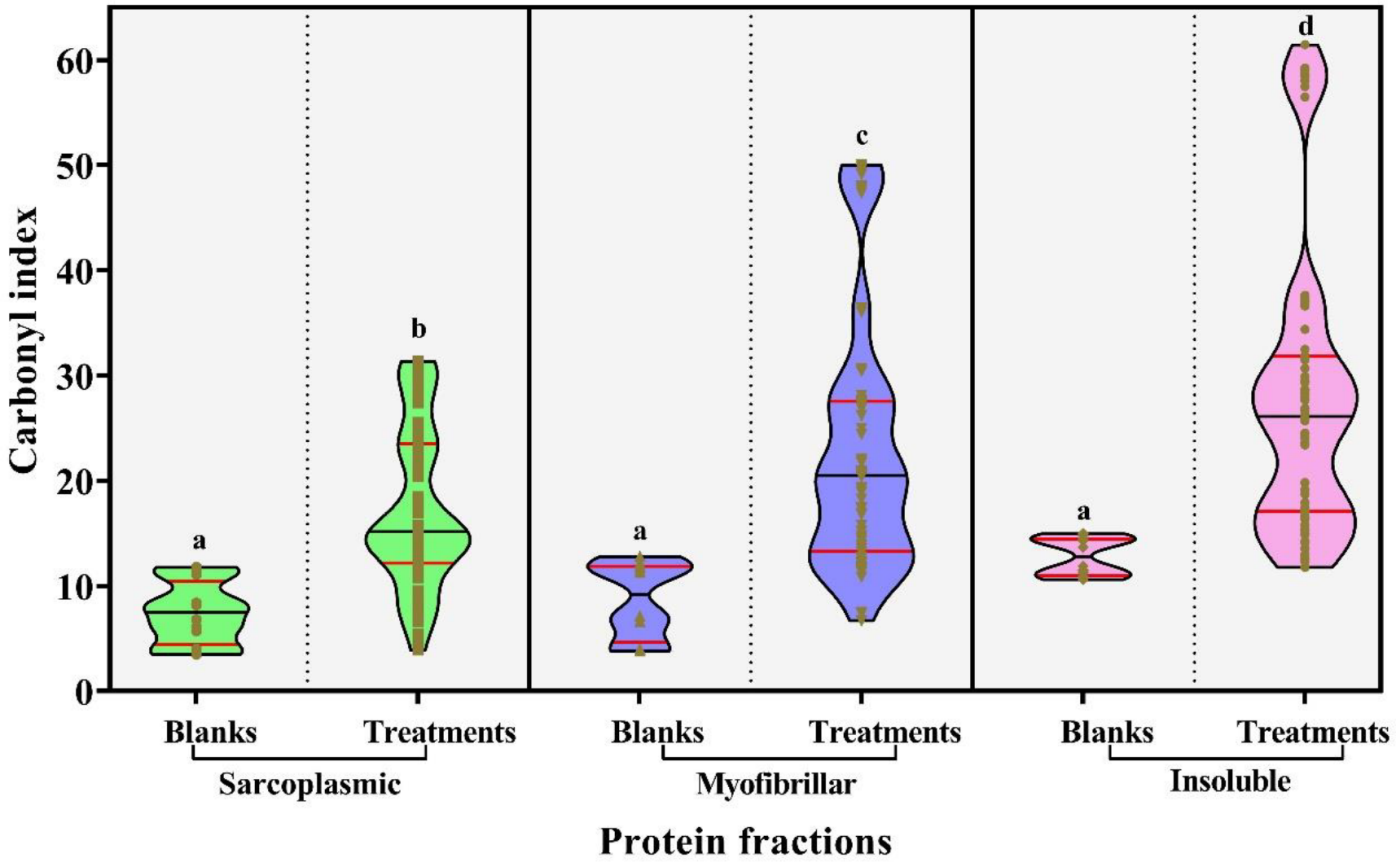
Carbonylation induced by assayed antibiotics (TCs and β-Lts) on main chicken breast proteins. The colors of the violins allude to the type of protein fraction: Green: sarcoplasmic proteins; Blue: myofibrillar proteins and Purple: insoluble proteins. The medians and quartiles are showed as black and red lines in the violin boxes, respectively. Different letters on the plots expressed significant differences in protein carbonylation between samples.

The oxidative capability of the assayed antibiotics was evaluated by determination of the carbonylation degree of exposed sarcoplasmic, myofibrillar and insoluble chicken breast proteins, and was expressed as the carbonyl index (CI) in nmol of carbonyls·mg ^-1^ of proteins. CIs found in the control samples were on average 7.40 ± 0.85; 8.67 ± 1.03 and 12.79 ± 0.5178 nmol of carbonyls·mg ^-1^ of proteins for sarcoplasmic, myofibrillar and insoluble, respectively. When those values were compared with the treated samples, it was observed that 16 of the 24 treatments assayed (66.7%) significantly increased their carbonyl groups, as shown in Figure 1.

The CIs induced by antibiotics on protein fractions were between 3.92 – 31.38; 6.72 – 50.07 and 11.77–61.49 nmol of carbonyls·mg ^-1^ of proteins for sarcoplasmic, myofibrillar and insoluble, respectively. In addition, the developed Kruskal-Wallis test among the CIs from the three proteins groups showed that there were significant differences between their medians ( *p<0.05*), the insoluble proteins being the ones that showed the highest CIs (28.04 ± 1.61 nmol of carbonyls·mg ^-1^ of proteins), followed by myofibrillar and sarcoplasmic proteins with mean values of 23.07 ± 1.42 and 17.28 ± 0.89 nmol of carbonyls·mg ^-1^ of proteins, respectively. Next, we described the behavior of induced carbonylation by each group of antibiotics.

### Behavior of TCs-induced carbonylation

**Figure 2.**
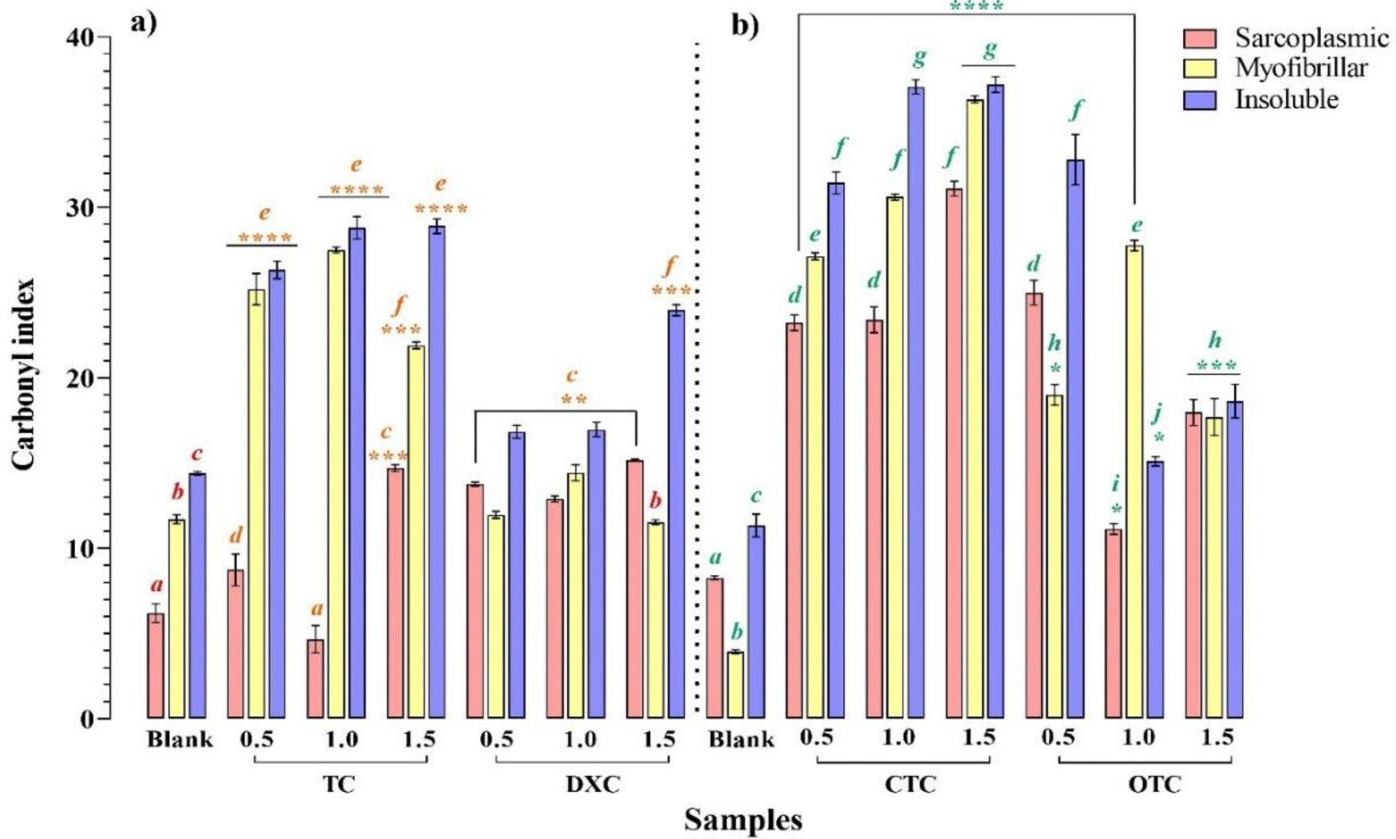
Changes in carbonyl content of sarcoplasmic, myofibrillar and insoluble chicken breast proteins induced by tetracycline (TC), chlortetracycline (CTC), oxytetracycline (OTC) and doxycycline (DXC). The columns in the graph are grouped as: a) TC and DXC treatment´s and their blanks b) CTC and OTC and their blanks. Mean values ± SEM. ^a-j^ Different letters on the bars denote statistically significant differences. Means having different superscripts differ between blank and tetracyclines concentration ( *p* < 0.05). **** *p* < 0.0001, *** *p* < 0.005, ** *p* < 0.05 denote statistically significant differences among treatment and their respective control.

TCs’ oxidative capability is shown in Figure 2. In sarcoplasmic proteins, all assayed concentrations of CTC, OTC and DXC induced a significant carbonylation compared to the control ( *p* < 0.05); in contrast, the samples contaminated with TC, only showed a significant increase at 0.5 and 1.5 MRL ( *p* < 0.05). The maximum oxidant power of TC, CTC and DXC was observed at 1.5 MRL, while OTC was set at 0.5 MRL. Contrarily, in myofibrillar proteins, TC, CTC and OTC promoted a significantly higher carbonylation compare to control ( *p* < 0.05) at all concentrations assayed. DXC only induced a significant carbonylation at 1.0 MRL regarding to the control ( *p* < 0.05). Unlike to sarcoplasmic proteins, the maximum oxidant power of TC, OTC and DXC was observed at 1.0 MRL, while CTC’s continued at 1.5 MRL ( *p* < 0.05).

Finally, for insoluble proteins, all concentrations of tetracyclines induced significant carbonylation with respect to control ( *p* < 0.05). The maximum carbonylation promoted by TC and CTC was observed at 1.0 and 1.5 MRL ( *p* > 0.05), while OTC and DXC were at 0.5 and 1.5 MRL, respectively.

### Behavior of β-Lts-induced carbonylation

**Figure 3.**
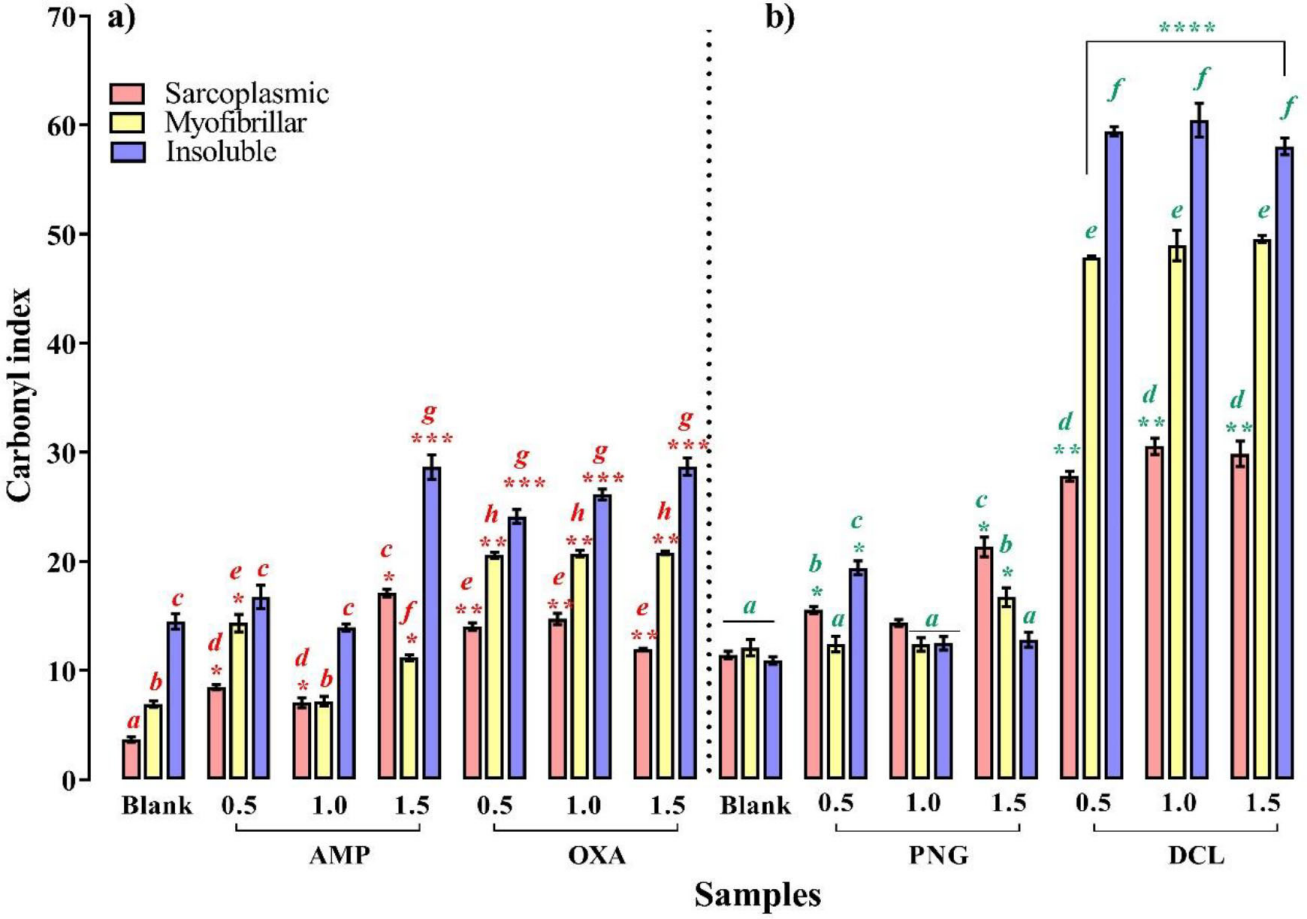
Changes in carbonyl content of sarcoplasmic, myofibrillar and insoluble chicken breast proteins induced by ampicillin (AMP), penicillin (PNG), oxacillin (OXA) and dicloxacillin (DCL). Mean values ± SEM. Letters ^a-j^ above the bars denote statistically significant differences in carbonylation compared to blank. Means having different superscripts differ between blank and tetracyclines concentration ( *p* < 0.05). **** *p* < 0.0001, *** *p* < 0.005, ** *p* < 0.05 denote statistically significant differences among treatment and their respective control.

β-Lts-induced carbonylation is shown in Figure 3. In sarcoplasmic proteins, all the assayed concentrations induced significant carbonylation on contaminated samples compared to control ( *p* < 0.05). For samples contaminated with AMP and PNG, maximum carbonylation was observed at 1.5 MRL, while in samples contaminated with DCL, maximum carbonylation was set at 1.0 MRL;for OXA the values were set at 0.5 and 1.0 MRL. There were no statistical differences between their CIs ( *p* > 0.05). In contrast, in myofibrillar proteins, DCL and OXA induced the same carbonylation in treated samples at all assayed concentrations ( *p* > 0.05), while AMP only induced a significant carbonylation at 0.5 and 1.5 MRL with respect to control ( *p* < 0.05), and PNG a 1.5 MRL carbonylation. Finally, for insoluble proteins, PNG, DCL and OXA promoted a significantly higher carbonylation than control at all concentrations assayed ( *p* < 0.05), while AMP only led to a 1.5 MRL carbonylation. For AMP and OXA, the maximum oxidant power was induced at 1.5 MRL, whereas that of DCL was similar for all three concentrations ( *p* > 0.05).

### Comparison between carbonylation induced by TCs and β-LTs

**Figure 4.**
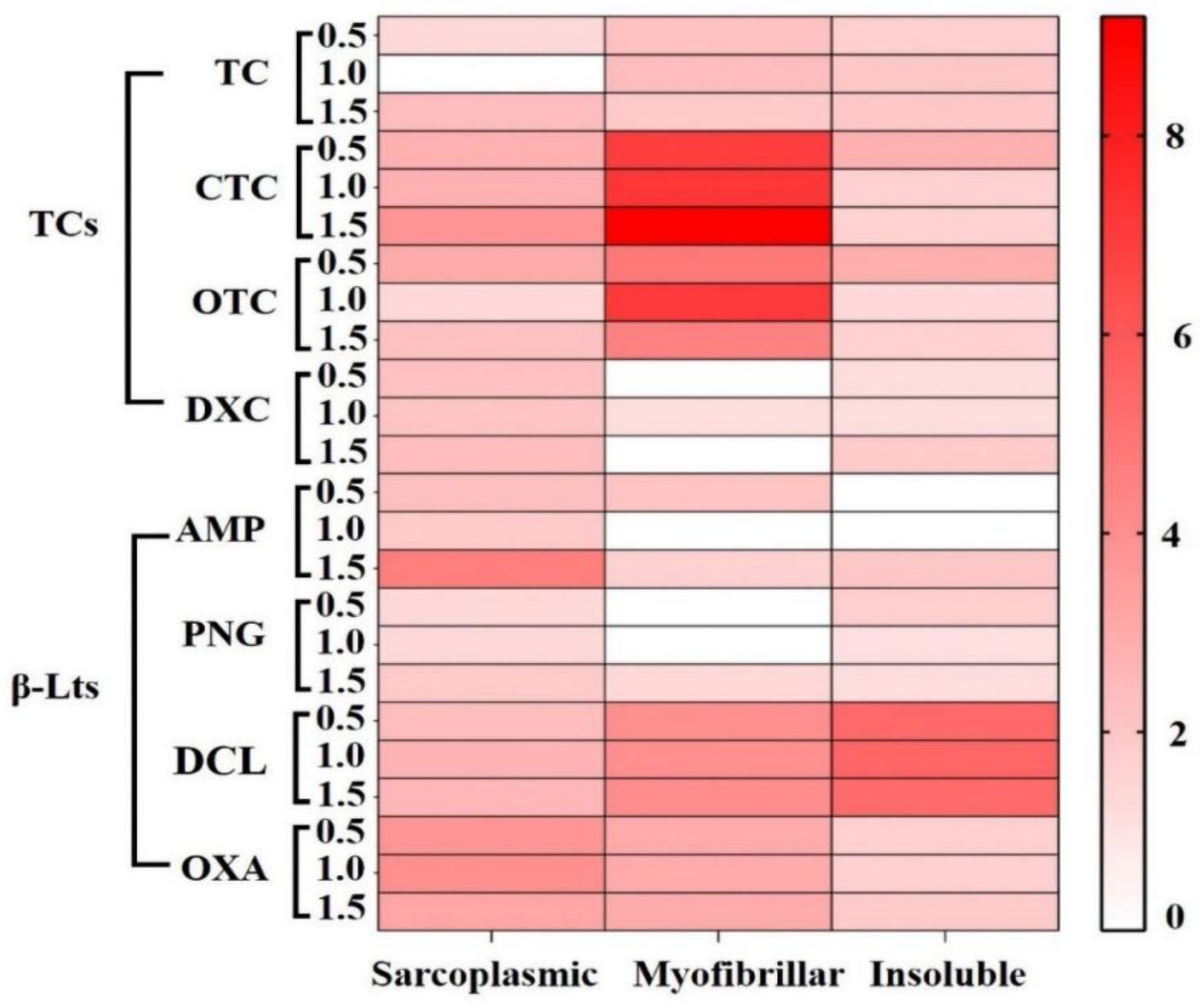
Heat map with average values of the oxidant power (ratios) induced by the evaluated TCs and β-LTs. The results of the comparative analysis between the carbonylation induced by TCs and β-Lts according to the type of fraction is described in the first item ( Figure 1). On the other hand, the medians of the carbonylation indices (CIs) promoted by the two groups of antibiotics evaluated through the Mann-Whitney test were compared; results showed that there were no significant differences between the CIs in the samples exposed to both groups of antibiotics ( *p* = 0.3714), Figure 5.

Figure 4 shows the oxidant power of TCs and β-LTs on sarcoplasmic, myofibrillar and insoluble proteins from chicken breast; oxidant power was calculated using the ratio between the CIs from treated samples and from the controls (Carbonyl index of protein fraction from contaminated samples/Carbonyl index of blank samples). As showed, among all antibiotics assayed, CTC and OTC promoted the highest carbonylation in myofibrillar proteins at all assayed concentrations (with average ratios of 7.9 and 5.4, respectively); these were followed by DCL in the insoluble (average ratio of 5.4) and myofibrillar fractions (average ratio of 4.0) and OXA in the sarcoplasmic and myofibrillar fractions (average ratios of 3.7 and 3 respectively). Oppositely, DXC, AMP and PNG induced the lowest carbonylation, mainly in myofibrillar (average ratios of 0.4, 0.5 and 0.5, respectively) and insoluble fractions (average ratios of 1.4, 0.7 and 1.4, respectively).

**Figure 5.**
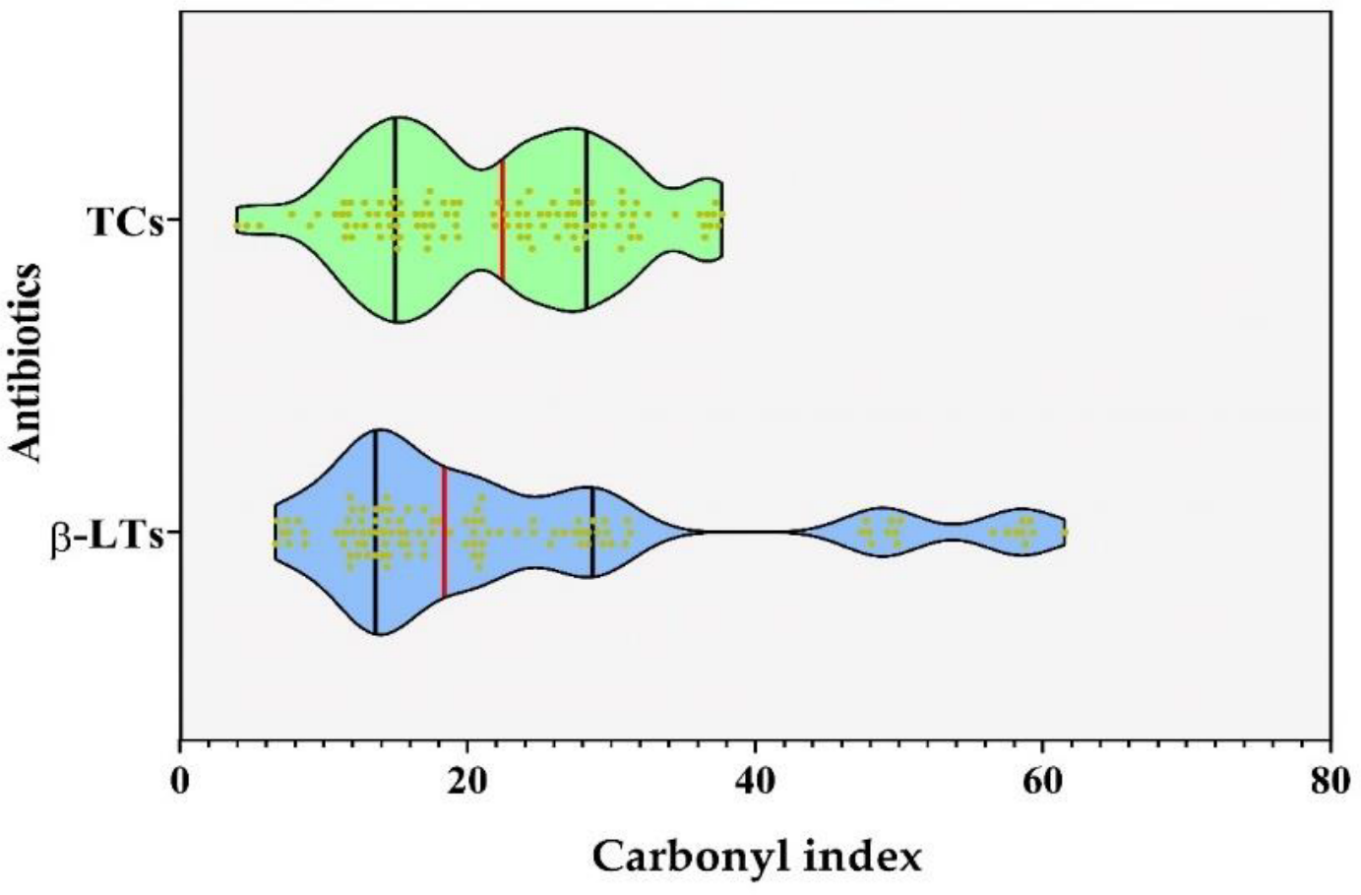
Carbonylation induced by assayed antibiotics (TCs and β-Lts) on main chicken breast protein fractions. The medians and quartiles are showed as black and red lines in the violin boxes, respectively.

According to the carbonylation variability induced by the assayed antibiotics, we decided to assess individual and multiple correlations among CIs and independent variables assessed in the study: *protein fraction, type of antibiotic, evaluated concentration of each antibiotic, and octanol-water partition coefficient (LogP).* This last variable was included given that the antibiotics evaluated, by belonging to the same group, have similar chemical structures (pharmacophore) differing only in their radicals; therefore one of the most used parameters to evaluate differences in their toxicological behaviors in *in vitro* models are their solubility variations (LogP). ^17^

**Figure 6.**
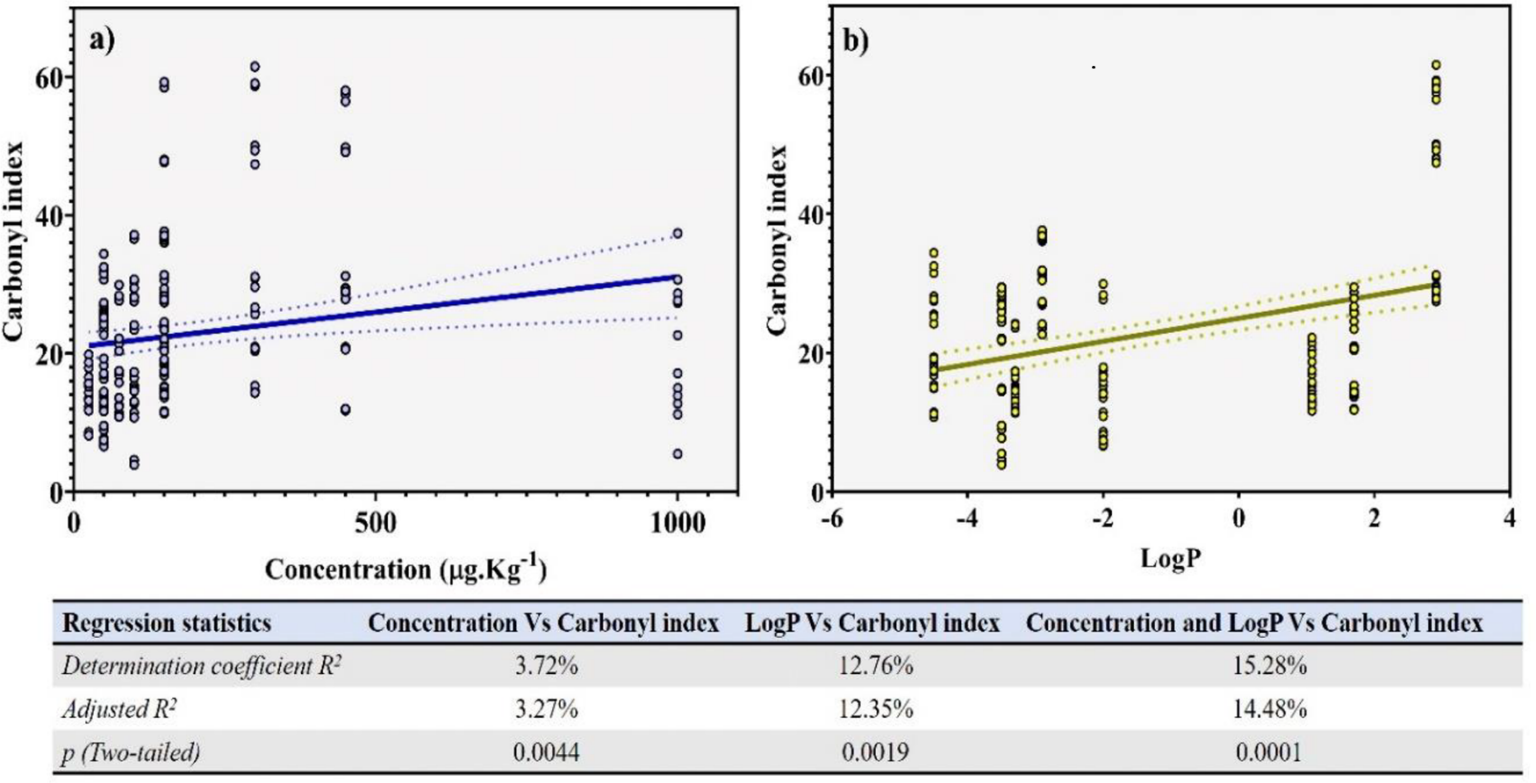
Multiple linear correlation analysis performed between antibiotic-induced carbonylation vs (a) assayed concentration of antibiotics and (b) antibiotics’ Partition Coefficient (log P). These parameters were used to evaluate the variables’ relation level were the correlation and determination coefficients.

Simple regression analysis results between CIs induced by antibiotics at their respective concentration, and LogP revealed a significantly positive relationship for both variables combinations (R ^2^ = 0.0327, *p* = 0.0044 for CIs and antibiotics concentrations, and R ^2^ = 0.1235, *p* = 0.0019, for CIs and LogP). This indicates that they represent important parameters in the oxidant properties of antibiotics ( Figure 6a and 6b). When comparing adjusted determination coefficients obtained in correlation analyses, we were able to establish a hierarchy in the effect exercised by the evaluated independent variables (concentration and LogP) on the antibiotics-induced carbonylation, showing the following increasing order: LogP > concentration. Nevertheless, in order to evaluate the joint correlation level of independent variables assayed (concentration and LogP) vs the antibiotic-induced carbonylation, a multiple linear regression analysis was performed. Thus, the comparison between adjusted R ^2^ obtained in the simple and multiple linear regression analysis showed that R ^2^ in combined variables (14.48%) was higher than that of individual ones (3.27% and 12.35% for concentration and LogP, respectively), thus attributing the antibiotic-induced carbonylation effect to the synergic effect of the evaluated independent variables ( *p* < 0.0001).

## Discussion

The results described before show the capability of TCs and β-LTs to promote the carbonylation of chicken breast proteins even at concentrations under their MRLs.

In addition, when comparing the carbonylation induced by tetracyclines in three different protein fractions, it can be observed that sarcoplasmic and myofibrillar proteins were the most oxidized ones ( Figure 4). This behavior could be associated to accessibility of proteins in muscle cells: sarcoplasmic being more available than myofibrillar, which are more available than insoluble proteins; this is due to sarcoplasmic proteins being soluble in sarcolemma, while myofibrillar and insoluble proteins are stored in myofibrilla and connective tissue, respectively. ^13, 18^

Finally, carbonylation of chicken breast proteins provides evidence for the ability of tetracyclines to promote oxidative stress, which is consistent with assays carried out with several biological models. ^19– 24^ According to Wen *et al.* (2012), CTC induced oxidative stress in maize root through the production of hydroxyl radicals ^19^; Pes *et al.* (2018) reported a decrease of antioxidant enzymes in fish when these were exposed to OTC; Hang *et al.* (2019) showed that exposure to TC induced oxidative stress in ryegrass seedlings mediated by the increase of lipid oxidation and decrease of antioxidants enzymes. ^9^ However, according to the literature, DXC has antioxidant properties, which is contradictory to our results. ^21– 23^ This fact is interesting since the concentrations assayed in this study were lower than the ones used in studies where the protecting effect against oxidation was observed (16 times approximately) ^21– 23^; maybe this behavior could be associated to endocrine-disrupting effects.

In a similar way as tetracyclines, these results show the capacity of β-lactams to induce oxidation in chicken breast proteins even at levels under their MRLs, and therefore their ability to induce oxidative stress. However, these results are contradictory to the literature, because β-lactams have been attributed antioxidant properties: Berczyński *et al.* (2017) demonstrated the antioxidant capacity of β-lactams in the following descending order: ampicillin, penicillin, dicloxacillin and finally oxacillin, against reactive oxygen species. ^24^ Although Dwyer *et al.* (2014), observed that some antibiotics, suchas ampicillin induced oxidative stress to increase their antibiotic lethality. Thus, β-lactam behavior could be bivalent (oxidant/antioxidant), explaining the lower levels of carbonylation induced of chicken breast proteins ( Figure 4). While comparing the carbonylation on three protein fractions, only DCL and OXA promoted greater oxidative damage on these ( Figure 4), probably mediated by a largest number of molecules of DCL and OXA available in the muscle cell, these amounting to 6 times the concentrations of PGN and AMP. ^4, 13^

According to the literature, the carbonylation induced by residues of tetracyclines and β-lactams at MRL concentrations are similar to those promoted by fluroquinolones MRLs on beef proteins, which was reported by Marquez *et al.* (2020). In this study, fluoroquinolones also induced carbonylation even at concentrations under MRL values, and related to solubility loss and decreased protein digestibility. ^13^ Therefore, it would be expected that these types of changes can occur in chicken muscle proteins as well and influence the meat’ nutritional value. ^14^

In conclusion, our results showed the oxidizing effect of tetracycline and β -lactam residues on chicken breast proteins, even at concentrations considered safe. This causes concern because carbonylation continues to occur even after the animal is slaughtered, which means that under *in vivo* conditions protein oxidation may be greater. This could also affect the nutritional value of meat proteins, since carbonylation causes solubility loss and decreased digestibility in proteins, as well as loss of protein functionality.

## Data availability

### Underlying data

Figshare: Raw data of chicken breast proteins exposure to antibiotics, https://doi.org/10.6084/m9.figshare.14799147.v3

This project contains the following underlying data:

• Data set 1: Raw data from carbonyl assay of tetracyclines.
• Data set 2: Raw data from carbonyl assay of β-lactams.
• Raw data Figures.
• Raw data Figures from GraphPad Prism.

Data are available under the terms of the Creative Commons Zero “No rights reserved” data waiver (CC0 1.0 Public domain dedication).
