# Supplementary material for: Residues of tetracyclines and β-lactams antibiotics induce carbonylation of chicken breast
Source: F1000Res. 2021 Jul 15;10:575. [Version 1] doi: 10.12688/f1000research.53863.1 (PMC8917320; doi:10.12688/f1000research.53863.1)
Supplement: Peer review history

# Peer review: Reviewer response for version 1

**DOI:** 10.5256/f1000research.57287.r121739

**Published:** 2022-03-11

**Authors:** Chenliang Li

**Affiliations:** 1 Instituto de Agroquímica y Tecnología de Alimentos (IATA-CSIC), Valencia, Spain

The main results of this study show that the presence of tetracyclines and β-lactams residues at MRLs concentrations promotes *in vitro *carbonylation on chicken breast proteins. The authors give an interesting insight into the impact of antibiotics on the integrity of meat proteins towards human consumption. This manuscript has been well-written and discussed in terms of the topic which has good novelty. This reviewer would thereby recommend it for indexing as it is.

 Some suggestions:

1. The third paragraph of the Introduction section: 'Despite...are one of its most abundant constituents' This sentence was too long, suggest to isolate it into two individual ones.
2.  In the section entitled 'Preparation of antibiotics solutions':change '...until obtaining an antibiotic concentration...' into '...to obtain an antibiotic concentration...'; change '...in order to avoid sample protein precipitation...' into '...to avoid protein precipitation...'
3. In section entitled with 'Behavior of TCs-induced carbonylation': change '...higher carbonylation compare to control (p < 0.05) at all concentrations assayed' into '...higher carbonylation compare to control (p < 0.05) regardless of concentrations assayed'
4. In the section entitled 'Comparison between carbonylation induced by TCs and β-LTs': change 'Thus, the comparison between adjusted R2 obtained in the simple and multiple linear regression analysis showed that...' into 'As a result, the comparison between...'
5. In the Discussion section: change '...; maybe this behavior could be associated to endocrine-disrupting effects.' into '..., which sheds light in endocrine-disrupting effects.'
6.  In the Discussion section: 'In this study, fluoroquinolones also induced carbonylation even at concentrations under MRL values, and related to solubility loss and decreased protein digestibility'. Please specify clearer which concentrations (with values) you mentioned.
7.  In the Discussion section: 'This could also affect the nutritional value of meat proteins since carbonylation causes solubility loss and decreased digestibility in proteins, as well as loss of protein functionality.' Change '...loss of protein functionality' into 'an alteration in protein (bio)availability and/or functionality'.

Is the work clearly and accurately presented and does it cite the current literature?

Yes

If applicable, is the statistical analysis and its interpretation appropriate?

Yes

Are all the source data underlying the results available to ensure full reproducibility?

Yes

Is the study design appropriate and is the work technically sound?

Yes

Are the conclusions drawn adequately supported by the results?

Yes

Are sufficient details of methods and analysis provided to allow replication by others?

Yes

Reviewer Expertise:

Meat science, protein oxidation, peptide chemistry, omics

I confirm that I have read this submission and believe that I have an appropriate level of expertise to confirm that it is of an acceptable scientific standard.

# Peer review: Reviewer response for version 1

**DOI:** 10.5256/f1000research.57287.r102491

**Published:** 2022-02-18

**Authors:** Małgorzata Korzeniowska

**Affiliations:** 1 Department of Functional Food Products Development, Faculty of Biotechnology and Food Sciences, Wrocław University of Environmental and Life Sciences, Wrocław, Poland

The manuscript is well written, based on a well-designed, reliable, and reproducible experiment. The subject of the study is interesting for readers and relevant to the discipline. 

 The abstract is informative and clearly states the most relevant findings. The introduction shows the main findings in the area and impact on chicken meat and possible influence on humans. The objective is short and straight to the point. The methodology is known and already established. It would be beneficial to have data on the respective antibiotic concentrations in the used material which was purchased in the local market. The lack of this information (analysis) is the main gap in the study. It would be great to have chicken meat collected directly from the farm to have the knowledge about real residues of the antibiotics in meat and to be able to draw a conclusion about the mechanism of action. Results and discussion are well prepared, leading to the conclusion. 

 The manuscript can be indexed in its current form.

Is the work clearly and accurately presented and does it cite the current literature?

Yes

If applicable, is the statistical analysis and its interpretation appropriate?

Yes

Are all the source data underlying the results available to ensure full reproducibility?

Yes

Is the study design appropriate and is the work technically sound?

Yes

Are the conclusions drawn adequately supported by the results?

Yes

Are sufficient details of methods and analysis provided to allow replication by others?

Yes

Reviewer Expertise:

Poultry meat quality

I confirm that I have read this submission and believe that I have an appropriate level of expertise to confirm that it is of an acceptable scientific standard.
